# Persistence of HCV in Acutely-Infected Patients Depletes C24-Ceramide and Upregulates Sphingosine and Sphinganine Serum Levels

**DOI:** 10.3390/ijms17060922

**Published:** 2016-06-13

**Authors:** Georgios Grammatikos, Julia Dietz, Nerea Ferreiros, Alexander Koch, Georg Dultz, Dimitra Bon, Ioannis Karakasiliotis, Thomas Lutz, Gaby Knecht, Peter Gute, Eva Herrmann, Stefan Zeuzem, Penelope Mavromara, Christoph Sarrazin, Josef Pfeilschifter

**Affiliations:** 1 Pharmazentrum Frankfurt, Institut für Allgemeine Pharmakologie, Goethe University Hospital, Frankfurt am Main, Theodor-Stern-Kai 7, 60590 Frankfurt am Main, Germany; Koch@med.uni-frankfurt.de (A.K.); pfeilschifter@em.uni-frankfurt.de (J.P.); 2 Medizinische Klinik 1, Goethe University Hospital, Frankfurt am Main, Theodor-Stern-Kai 7, 60590 Frankfurt am Main, Germany; julia.dietz@em.uni-frankfurt.de (J.D.); georg.dultz@kgu.de (G.D.); zeuzem@em.uni-frankfurt.de (S.Z.); sarrazin@em.uni-frankfurt.de (C.S.); 3 Pharmazentrum Frankfurt, Institut für klinische Pharmakologie, Goethe University Hospital, 60590 Frankfurt am Main, Germany; ferreirosbouzas@em.uni-frankfurt.de (N.F.); Herrmann@med.uni-frankfurt.de (E.H.); 4 Institute of Biostatistics and Mathematical Modelling, Department of Medicine, Goethe University, 60590 Frankfurt am Main, Germany; bon@med.uni-frankfurt.de; 5 Molecular Virology, Hellenic Pasteur Institute, 11521 Athens, Greece; karakasiliotis@pasteur.gr (I.K.); penelopm@pasteur.gr (P.M.); 6 Infektiologikum, 60590 Frankfurt am Main, Germany; lutz@infektiologikum.de (T.L.); knecht@infektiologikum.de (G.K.); gute@infektiologikum.de (P.G.); 7 Molecular Biology and Genetics, Democritus University of Thrace, 68100 Alexandroupolis, Greece

**Keywords:** HIV, hepatitis C, sphingolipid, biomarker, angiopoietin-like 3 (ANGPTL3)

## Abstract

Hepatitis C virus (HCV) substantially affects lipid metabolism, and remodeling of sphingolipids appears to be essential for HCV persistence *in vitro*. The aim of the current study is the evaluation of serum sphingolipid variations during acute HCV infection. We enrolled prospectively 60 consecutive patients with acute HCV infection, most of them already infected with human immunodeficiency virus (HIV), and serum was collected at the time of diagnosis and longitudinally over a six-month period until initiation of antiviral therapy or confirmed spontaneous clearance. Quantification of serum sphingolipids was performed by liquid chromatography-tandem mass spectrometry (LC-MS/MS). Spontaneous clearance was observed in 11 out of 60 patients (18.3%), a sustained viral response (SVR) in 43 out of 45 patients (95.5%) receiving an antiviral treatment after follow-up, whereas persistence of HCV occurred in six out of 60 patients (10%). C24-ceramide (C24-Cer)-levels increased at follow-up in patients with spontaneous HCV eradication (*p* < 0.01), as compared to baseline. Sphingosine and sphinganine values were significantly upregulated in patients unable to clear HCV over time compared to patients with spontaneous clearance of HCV infection on follow-up (*p* = 0.013 and 0.006, respectively). In summary, the persistence of HCV after acute infection induces a downregulation of C24Cer and a simultaneous elevation of serum sphingosine and sphinganine concentrations.

## 1. Introduction

Acute infection with hepatitis C virus (HCV) remains in most cases clinically asymptomatic. The persistence of HCV occurs in approximately 75% of cases [1], and recent studies observed an epidemic transmission of HCV in injecting drug users (IDU) and in men having sex with men (MSM), mostly on the background of a chronic infection with human immunodeficiency virus (HIV) [2]. Since clinical management of acute HCV infection is still a matter of debate despite the availability of direct-acting antivirals (DAAs), the prediction of potential spontaneous clearance and identification of the optimal time point for therapeutic intervention require further insights into translational modifications occurring during acute HCV infection.

Numerous basic research studies highlighted the dependence of HCV on host lipid metabolism [3,4,5,6]. Particularly, sphingolipids (SLs), a diverse complex class of sphingosine-derived lipids with pleiotropic effects on the pathogenesis of various diseases, have been shown to affect replication and persistence of HCV *in vitro* [7,8,9]. Especially ceramide (Cer), the hydrophobic and functional backbone of SLs, alters the biophysical properties of biological membranes [10] and, thus, regulates the entry and replication of HCV in the host cell [11,12]. NS5B polymerase, which is crucial for HCV replication, possesses a sphingomyelin binding domain [13] and is critically affected by SL-regulated lipid peroxidation [14]. Additionally, inhibition of enzymes regulating the SL metabolism modifies the replication of HCV *in vitro* [14]. Yet, the interaction of HCV with lipids appears to be reciprocal, since chronic HCV infection is known to induce hypocholesterolemia [15]. Moreover, the expression of angiopoietin-like 3 (ANGPTL3), a liver-secreted protein able to affect serum lipid concentrations by regulation of serum lipases [16], was also shown to be negatively regulated by HCV [17]. HCV infection modulates ANGPTL3 levels by repression of hepatic nuclear factor (HNF) 1α [17], which is itself downregulated by Cer [18]. Yet, a direct interaction between ANGPTL3 and SLs has not been described so far. Regarding the role of lipids as potential biomarkers, serum lipids and apolipoproteins correlate with the efficiency of antiviral treatment [19,20], and plasma apolipoprotein H levels are associated with spontaneous clearance of HCV during acute infection [21].

In our own, recent studies, we identified significant translational variations of the SL profile in chronic HCV infection. Particularly in the serum of patients chronically infected with HCV, we observed a significant upregulation of secreted acid sphingomyelinase activity [22]. Furthermore, chronic HCV infection induced a marked downregulation of C24Cer [22], and low C24Cer levels were in consecutive studies associated with severe liver fibrosis and poor responsiveness to antiviral therapy [23], as well as with clinical deterioration of HCV-associated cirrhosis [24]. Further SL metabolites, such as dihydroceramides (DHCs), able to regulate autophagy [25], correlated in our observations with the levels of aminotransferases [23], while sphingosine and sphinganine were identified in a multivariate model as novel independent predictors of severe HCV fibrosis [23].

The purpose of the current study is therefore the evaluation of serum SL concentrations during acute HCV infection to identify potential variations of serum SL levels upon spontaneous clearance or persistence of HCV. We further assessed potential correlations of ANGPTL3 levels to the outcome of acute HCV infection, and we also compared SL metabolite variations to already identified predictors of spontaneous clearance of HCV, such as the interleukin 28B (IL28B) genotype, HCV viral load, HCV genotype and to common biochemical markers in acutely-infected patients.

## 2. Results

### 2.1. Patient Characteristics

In the present prospective, study 60 consecutive patients with acute HCV infection were enrolled as mentioned above. The majority of patients were male (93.4%), chronically infected with HIV (85%) and acutely infected with HCV genotype 1 (73.4%). Patient characteristics are demonstrated in Table 1. Our analysis focused on three different patient groups depending on the outcome of acute HCV infection. Group A included patients with spontaneous clearance of acute HCV infection; Group B included patients who received antiviral treatment; and Group C included patients with persistence of HCV. Spontaneous clearance was observed in 11 out of 60 patients (18.3%), and 43 out of 45 patients (95.5%) receiving an antiviral treatment achieved an SVR. Forty of the treated patients received a combination of PEGylated (PEG)-interferon (IFN) and ribavirin (RBV) according to the respective clinical recommendations [26], while four patients received PEG-IFN-monotherapy, and one patient received PEG-IFN/RBV and telaprevir within an open label trial. The persistence of HCV infection was observed in six patients (10%). Two of these patients showed a non-response on antiviral treatment, and four patients did not receive antiviral therapy due to insufficient adherence and non-compliance.

### 2.2. Correlation of Serum Sphingolipids (SLs) with Hepatitis C Virus (HCV)/Human Immunodeficiency Virus (HIV) Coinfection, Angiopoietin-Like 3 (ANGPTL3) and Biochemical Parameters

**Figure 1:**
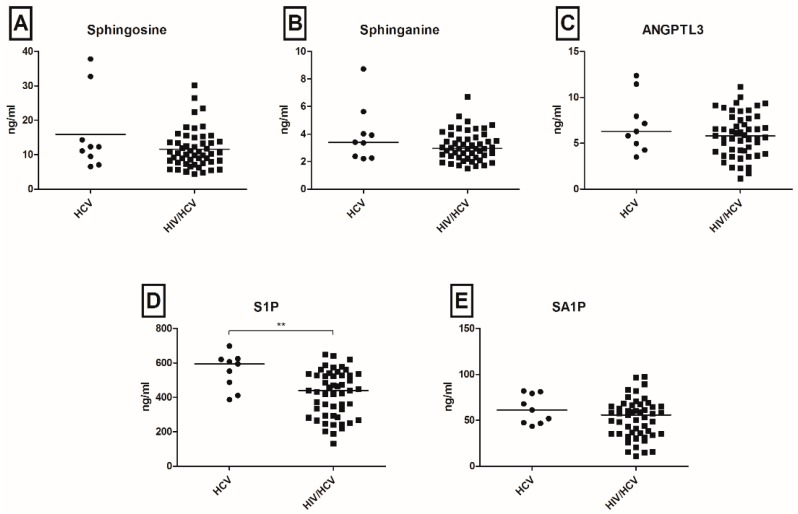
Serum sphingolipids (SLs) and angiopoietin-Like 3 (ANGPTL3) in hepatitis C virus (HCV)-mono-infection and in HCV/human immunodeficiency virus (HIV)-coinfection at baseline. S1P levels in HCV/HIV-coinfected patients were lower than in HCV-mono-infected patients ((**D**), ** *p* < 0.01) at baseline. No significant differences in the levels of further SL metabolites (**A**,**B**,**E**) and ANGPTL3 (**C**) were observed among mono- and co-infected patients. S1P: sphingosine 1-phosphate; SA1P: sphinganine 1-phosphate; ANGPTL3: angiopoietin-like 3.

To evaluate the effect of HCV/HIV coinfection on the sphingolipidomic (SLomic) profile, we compared SL levels in HCV mono-infected and HCV/HIV co-infected patients at baseline. Except from a significant reduction of S1P levels in HCV/HIV coinfection (*p* < 0.01, Figure 1D), no significant differences in the levels of further SL metabolites were observed among mono- and co-infected patients, both at baseline, as well as at follow-up (Figure 1, Supplementary Figures S1–S5). When common biochemical markers were compared to SL concentrations, the levels of Cer and DHC species correlated to the levels of transaminases, whereas solely S1P showed a significant inverse correlation to HCV viral load (Supplementary Table S1). Moreover, the evaluation of ANGPTL3 levels, a liver-secreted protein able to affect serum lipid concentrations, revealed neither an association with outcome of acute HCV infection (Supplementary Figure S6) nor a correlation with serum SL concentrations, apart from a weak positive association with C18Cer levels (Supplementary Table S1).

### 2.3. Spontaneous Clearance of HCV Restores Serum C24-Ceramide (C24-Cer) Levels

**Figure 2:**
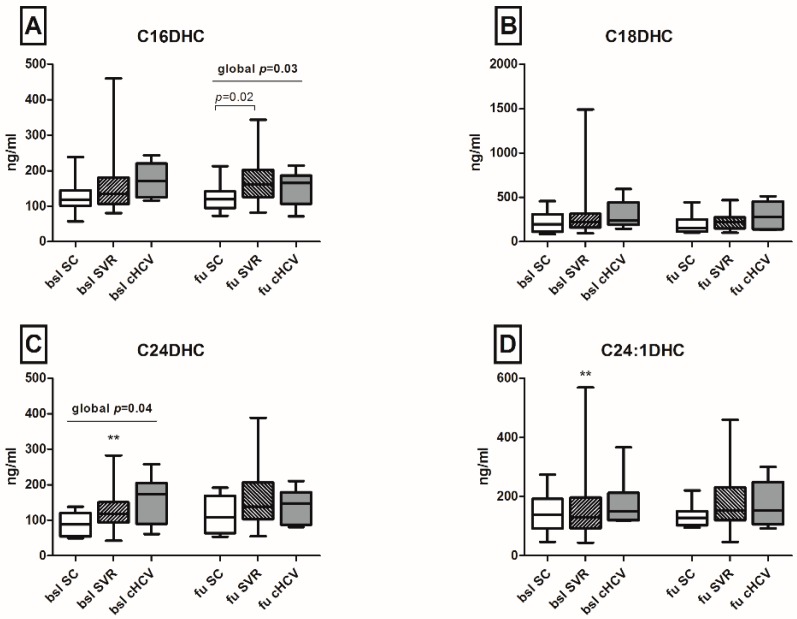
Serum dihydroceramide concentrations during acute HCV infection. C16DHC levels on follow-up were significantly lower in patients with spontaneous HCV clearance ((**A**), *p* = 0.02), while levels of C24DHC and of its saturated derivative C24:1DHC increased longitudinally in patients with subsequent SVR after antiviral treatment ((**C**,**D**), respectively, ** *p* < 0.01). No significant variations of C18DHC levels were observed both between patient groups and between baseline and follow-up (**B**). Statistical significance between baseline and follow-up is indicated by asterisks (*). DHC: dihydroceramide; SC: spontaneous clearance; SVR: sustained viral response; BSL: baseline; FU: follow-up; cHCV: chronic HCV.

**Figure 3:**
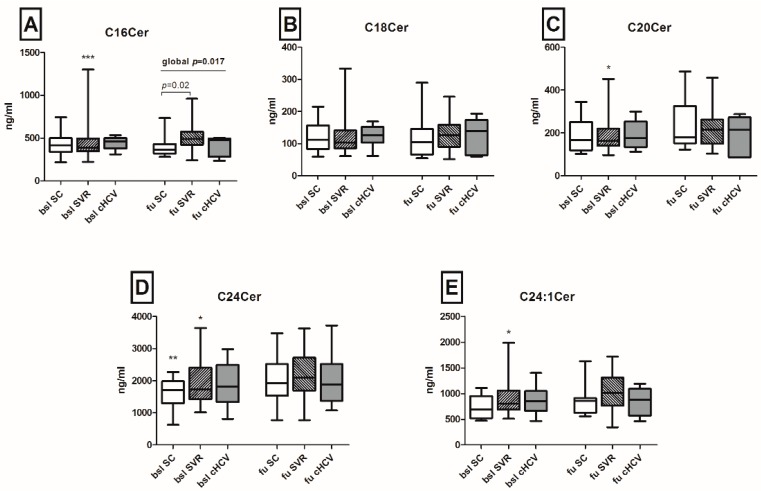
Serum ceramide variations during acute HCV infection. C16Cer, C18Cer, C20Cer, C24Cer and C24:1Cer levels increased during the course of untreated acute HCV infection in patients with subsequent SVR after antiviral therapy ((**A**) *** *p* < 0.001, (**B**) non-significant, (**C**) * *p* < 0.05, (**D**) * *p* < 0.05, ** *p* < 0.01 and (**E**) * *p* < 0.05, respectively). C24Cer levels increased significantly during the course of acute infection also in patients with spontaneous clearance, as well ((**D**), *p* < 0.01), and on follow-up, C16Cer levels were significantly lower in patients with spontaneous HCV clearance as compared to patients with subsequent SVR ((**A**), *p* = 0.02). Statistical significance between baseline and follow-up is indicated by asterisks (*). Cer: ceramide; SC: spontaneous clearance; SVR: sustained viral response; BSL: baseline; FU: follow-up; cHCV: chronic HCV.

Serum levels of DHCs and Cers with distinct fatty acid chain lengths were assessed in the patient cohort. Variations of serum SL metabolites were evaluated both among different outcomes (persistence and spontaneous or therapy-induced clearance of HCV), as well as longitudinally between baseline and follow-up for each outcome group. A significant increase in the levels of C24DHC and of its saturated derivative C24:1DHC was observed in patients with subsequent SVR after antiviral treatment in the longitudinal analysis (130 *versus* 162.1 and 160.9 *versus* 175.4 ng/mL between baseline and follow-up respectively, *p* < 0.01; Figure 2). Further, the analysis of Cer species indicated that C16Cer, C20Cer and C24:1Cer levels increased longitudinally in patients with subsequent SVR after antiviral therapy (*p* < 0.001, *p* < 0.05 and *p* < 0.05, respectively; Figure 3). C24Cer levels increased significantly during the course of acute infection in patients with spontaneous clearance (*p* < 0.01) or subsequent SVR after antiviral therapy (*p* < 0.05), while no differences were observed in patients with persistence of the virus (Figure 3D).

### 2.4. Upregulation of Sphingosine and Sphinganine Levels upon the Persistence of HCV

**Figure 4:**
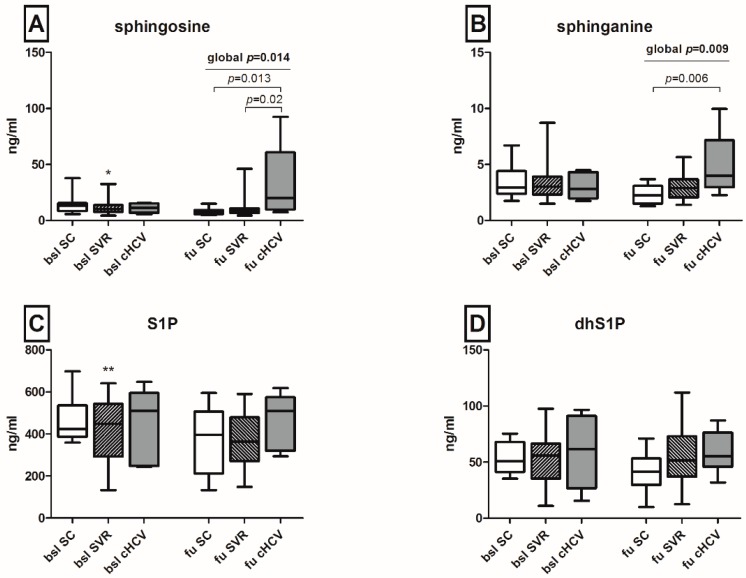
Sphingosine, sphinganine and their phosphate derivatives during acute HCV infection. Sphingosine levels on follow-up were significantly upregulated in patients unable to clear HCV compared both to patients with spontaneous or subsequent therapy-induced resolution of HCV infection ((**A**), *p* = 0.02 and *p* = 0.013, respectively). Sphinganine levels were significantly elevated upon chronification of HCV infection on follow-up ((**B**), *p* = 0.006), while patients with subsequent SVR after antiviral treatment showed a significant longitudinal reduction of S1P levels ((**C**), ** *p* < 0.01). No significant difference in dhS1P levels was observed (**D**). Statistical significance between baseline and follow-up is indicated by asterisks (*). S1P: sphingosine 1 phosphate; dh: dihydro; SC: spontaneous clearance; SVR: sustained viral response; BSL: baseline; FU: follow-up; cHCV: chronic HCV.

Sphingosine values were significantly upregulated in patients unable to clear HCV as compared both to patients with spontaneous (*p* = 0.013) or therapy-induced viral eradication (*p* = 0.02) of HCV at follow-up, while at baseline, no significant variations between the outcome groups were observed (Figure 4A). Similarly, sphinganine levels were significantly elevated upon chronification of HCV infection as compared to spontaneous clearance of the virus (*p* = 0.006) (Figure 4B). Regarding the corresponding phosphates, patients with achieved SVR after subsequent antiviral treatment showed a significant longitudinal reduction of S1P levels (*p* < 0.01, Figure 4C), while no significant variations in the levels of dhS1P were observed (Figure 4C).

### 2.5. Univariate Analysis of Baseline Markers Associated with Spontaneous HCV Clearance

To address the hypothesis of whether baseline SL concentrations are able to predict spontaneous clearance of HCV, we performed a univariate analysis of baseline parameters in patients with spontaneous clearance and in patients with persistence of HCV, as shown in Table 2. No significant differences between the two outcome groups were identified in the levels of aminotransferases, baseline viral load, bilirubin, thrombocytes, international normalized ratio (INR), as well as in the existence or not of an HIV-coinfection or of a favorable *IL28B-rs12979860* CC genotype (Table 2). Adequate HIV treatment, represented by the proportion of patients with low HIV viral load and by the count of CD4 cells, did not affect significantly the spontaneous clearance of HCV infection, as well (Table 2). The HCV genotype 1 and high C24DHC levels were the only parameters where a trend and significance were observed in patients with persistence of HCV as compared to patients with spontaneous clearance of the virus (*p* = 0.056 and 0.047, respectively).

## 3. Discussion

The pathogenesis and persistence of HCV infection are associated with modifications of the host lipid metabolism. Especially replication, particle assembly and exocytosis of HCV rely on SLs [7,8,14], a diverse class of lipids affecting several signaling pathways. Recently, we identified significant alterations of the SLomic profile in patients with chronic HCV infection and observed critical associations to the stage of HCV-induced liver fibrosis and decompensation of HCV-induced cirrhosis [22,23,24]. However, no data are available so far concerning early translational modifications of serum SLs occurring during the course of acute HCV infection.

Since most of the patients of our cohort were co-infected with HIV and SLs offer the essential metabolic environment for HIV fusion [27], entry [28] and escape from the host immune system [29], we compared the SLomic profile in HCV mono-infection and in HCV/HIV coinfection in order to exclude variations of serum SLs due to HIV infection. Except from significantly lower S1P levels in HCV/HIV coinfection, HIV infection did not affect the SLomic profile. Similarly, we evaluated the serum levels of ANGPTL3, a lipase secreted by the liver, which has been shown to affect HCV pathophysiology *in vitro* [17], and tested for potential correlations to serum SL concentrations and to the outcome of acute HCV infection. Despite the potential cross-talk between ANPTL3 and SLs via the interaction with HNF1α [17,18], in our current study, no significant associations were observed.

Interestingly, we observed in the present study a restoration of serum C24Cer levels in patients with spontaneous clearance or subsequent SVR after antiviral treatment, while the persistence of HCV maintained low levels of C24Cer in the respective patients. This finding is in line with our own recently published observations revealing a substantial translational implication of C24Cer in chronic HCV infection. Particularly, we have previously demonstrated that chronic HCV infection induces a marked downregulation of serum C24Cer concentrations [22] and low serum C24Cer levels are associated with severe liver fibrosis, poor responsiveness to antiviral therapy [23] and, in cirrhotic HCV patients with decompensation of liver cirrhosis, the occurrence of ascites and poor overall survival [24]. In the current study, while a significant increase of C24Cer levels was determined after spontaneous clearance of HCV (1631.4 at baseline *versus* 2052.8 ng/mL at follow-up, *p* < 0.01), serum concentrations of C24Cer were altogether lower compared to C24Cer levels in patients with chronic HCV infection (3280.4 ng/mL) and in healthy probands (4225.3 ng/mL), as observed by our group in a previous study [22]. A prolonged follow-up of patients regarding their serologic C24Cer levels after eradication of HCV and further basic research studies are needed to clarify the significance of these observations.

From a mechanistic point of view, animal model studies have described a severe hepatopathy as a consequence of genetic depletion of Cer-synthase 2, an enzyme predominantly expressed in the hepatic and renal tissue synthesizing C22-C24Cers [30]. Loss of Cer-synthase 2 activity was also linked to the promotion of chronic oxidative stress due to disruption of the mitochondrial respiratory chain [31]. Since HCV persistence is well known to be tightly linked to the regulation of oxidative stress and lipid peroxidation [14,32,33], the observed downregulation of C24Cer levels may constitute an additional link between viral persistence and SL-mediated regulation of oxidative stress by HCV. Furthermore, the ongoing characterization of distinct functions of Cers according to the length of the fatty acid attached to the sphingosine backbone has attributed pro-proliferative effects to C24Cer in contrast to the anti-proliferative effects of Cers with a shorter fatty acid chain [34]. Thus, HCV-induced liver injury due to direct cytopathic effects mediated by the virus through perturbation of the cell cycle [35] could also be partially induced by the ablation of C24Cer levels. In this context, we additionally identified an inverse correlation between bilirubin, AST and C24Cer in our patient cohort. Thus, our findings confirm the aforementioned observations of *in vitro* studies and offer valuable insights into early modifications of the SL metabolism during acute HCV infection on a translational level. Additional studies are needed to unravel the mechanistic background of these observations.

Further insights into the regulatory role of SLs in HCV infection have been recently provided by Yamane *et al.*, who identified a critical involvement of sphingosine-kinases in the replication process of HCV *in vitro* [14]. Inhibitors of regulatory SL enzymes induced an abrogation of HCV replication, while supplementation of the infectious HCV in cell culture (HCVcc) *in vitro* model with sphingosine and sphinganine, but not with S1P, promoted the replication of HCV strains [14]. In our current study, we observed a significant elevation of both sphingosine and sphinganine in patients with HCV persistence, as compared to spontaneous or therapy-induced clearance of the virus at follow-up. Since sphingosine has been long known to promote apoptosis and autophagy [36] and the occurrence of a severe hepatopathy in CerS2-knockout mice has been shown to correlate significantly with elevated sphinganine levels [30], both sphingosine and sphinganine may play a pivotal role in HCV-induced hepatopathy. Our observations on the upregulation of sphingosine and sphinganine six months after initial contact with the virus principally underline the early involvement of SLs in the process of HCV persistence and emphasize the potential utility of sphingosine and sphinganine as possible markers for the persistence of HCV during acute infection.

Although offering promising results, our study has some limitations. Due to the clinically unapparent course of acute HCV infection, only a moderate cohort size was included. Moreover, serum SL concentrations prior to acute HCV infection were missing, and further SLs, such as sphingomyelin, were not available in our current analysis and could thus not be included in the interpretation of our observations. Furthermore, the well-established translational link between insulin resistance and both HIV infection [37], as well as responsiveness to antiviral therapy in chronic HCV infection [38] implies a major role for insulin resistance in HIV/HCV coinfected patients. Additionally, serum sphingolipids have been shown to reflect insulin resistance in patients under physical exercise [39]. Unfortunately, data on insulin resistance were lacking in our patient cohort. In spite of these limitations, our results offer a significant advance over previous studies.

## 4. Experimental Section

### 4.1. Patients with Acute HCV Infection

In a prospective study, blood was collected from 60 consecutive patients with an acute HCV infection. Most patients were coinfected with HIV (51 out of 60, confirmed by a positive HIV antibody test), while 9 out of 60 patients were mono-infected with HCV. Diagnosis was performed at the University Hospital and the Infectious-Disease Medical Center (Infektiologikum) in Frankfurt am Main, Germany, between 2009 and 2013, as previously reported by our group [40]. Acute hepatitis C was in most cases diagnosed within the routine laboratory examination of HIV-infected patients, and diagnosis was defined by previously published criteria [41], such as the detection of HCV-RNA with or without concomitant positive anti-HCV antibodies in combination with a negative test for HCV-RNA or anti-HCV antibody in the last 12 months, as well as HCV-RNA positivity and elevation of the alanine aminotransferase (ALT) of more than 5-times the upper limit of the normal range and normal ALT within the last 12 months. Serum was collected at the time of diagnosis of acute HCV infection (termed as baseline) and longitudinally over a 6-month period until initiation of antiviral therapy or confirmed spontaneous clearance (termed as follow-up), as mentioned before [40]. The time point of acute hepatitis C infection was determined according to the medical history of each patient or, if not applicable, the time point of infection was estimated by calculating the mean date between the last time point with normal aminotransferases and the first time point with a positive HCV-RNA, as mentioned before [40]. Spontaneous clearance was defined as negative HCV-RNA detected twice within an interval of at least 12 weeks without receiving antiviral therapy and confirmation 6 months after diagnosis of acute hepatitis C. Sustained virologic response (SVR) was determined by HCV-RNA negativity 12 weeks after the end-of-treatment. Chronic HCV infection was defined as the presence of HCV RNA 24 weeks after diagnosis without treatment. One patient was excluded from the study due to being lost to follow-up. The study was performed in accordance with the Declaration of Helsinki and was approved by the local ethics committee. All patients had signed a written informed consent before study inclusion.

### 4.2. Determination of Sphingolipid Concentrations by High-Performance Liquid Chromatography Tandem Mass Spectrometry

Quantification of serum SLs was performed by high-performance liquid chromatography-tandem mass spectrometry (LC-MS/MS, AB Sciex, Darmstadt, Germany), as previously described [23]. For quantitation of SLs, 20 µL of serum were extracted with methanol/chloroform/HCl (15:83:2).

Afterwards, amounts of C16:0Cer, C18:0Cer, C20:0Cer, C24:0Cer, C24:1Cer, C16:0DHC, C18:0DHC, C24:0DHC, C24:1DHC and the internal standard (C17:0Cer) and sphingosine, sphingosine 1-phosphate (S1P) and sphinganine 1-phopshate (dhS1P) and the internal standards (sphingosine-D7, sphinganine-D7 and sphingosine 1-phosphate-D7) were analyzed by LC-MS/MS. All serum samples were stored at −80 °C until assayed. Further details on the mass spectrometric quantification of sphingolipids are provided in the Supplementary Material.

### 4.3. Detection of ANGTL3 Serum Concentrations

ANGPTL3 serum levels were determined by using the ANGPTL-3 ELISA kit (Adipogen AG, Liestal, Switzerland) according to the manufacturer’s instructions, as previously described [17].

### 4.4. Statistical Analysis

Statistical analysis for the scatter and box plots presented was performed with GraphPad Prism for Windows (v5.01; GraphPad Software Inc., San Diego, CA, USA). We carried out statistical analyses of the data using the BIAS software (BIAS for Windows, Version 10.11, Epsilon-Verlag, Darmstadt, Germany). Statistical comparisons for continuous variables with the outcome of acute hepatitis C were performed using the Wilcoxon–Mann–Whitney *U*-test (comparison of 2 groups) or the Kruskal–Wallis test (comparison of 3 or more groups). Dichotomic variables were assessed by means of contingency tables (Mantel–Haenszel’s test), as appropriate. The data are expressed as means ± standard error, unless otherwise specified. For statistical differences within the same group of patients over distinct time points, the Wilcoxon matched pairs test was applied. The level of significance was set at α = 0.05, representing the 95% confidence interval (CI). Statistically-significant differences are indicated in the corresponding figures (* *p* < 0.05; ** *p* < 0.01; *** *p* < 0.001).

## 5. Conclusions

In conclusion, our study emphasizes the potential of serum SLomics as a promising approach to identify novel biomarkers of acute HCV infection. Particularly, the serologic determination of C24Cer, sphingosine and sphinganine during the first months of acute HCV infection may contribute to the correct identification of patients being at a higher risk for developing a chronic infection and, thus, suitable for early initiation of antiviral therapy. Further prospective studies on the SL profile of the host during acute HCV infection are required for the implementation of SLs as biomarkers for clinical decision making.

## Figures and Tables

**Table 1:** Patient’s baseline biochemical and virological characteristics.

Parameters	Patients with Acute HCV Infection (*n* = 60)
Age, years: mean (range)	42 (22–61)
Sex, male, *n* = (%)	56 (93.4)
Female, *n* = (%)	4 (6.6)
ALT, IU/L, mean (range)	505 (28–2738)
AST, IU/L, mean (range)	242 (21–1957)
γGT, IU/L, mean (range)	250 (25–1064)
Bilirubin, mg/dL, mean (range)	1.0 (0.2–13)
Thrombocytes, cells/µL, mean (range)	210 (115–324)
INR, mean (range)	1.05 (0.9–1.3)
IL28B-rs12979860 genotype, (CC/non_CC, *n* = (%))	26 (43.4)/34 (56.6)
HCV genotype	
1, *n* =	44
1a, *n* =	39
1b, *n* =	4
Subtype not determined, *n* =	1
2, *n* =	2
3, *n* =	3
4, *n* =	11
HCV viral load, IU/mL: mean (range)	7.7 × 10^6^ (15–(8.6 × 10^7^))
Estimated duration between infection to diagnosis (weeks) *: mean (range)	7 (1–20)
Duration diagnosis to initiation of therapy (weeks): mean (range)	14 (3–33)
Duration diagnosis to spontaneous clearance (weeks): mean (range)	30 (4–100)
Spontaneous clearance, *n* = (%)	11 (18.3)
Antiviral therapy of acute HCV, *n* = (%)	45 (75)
SVR, *n* = (% of follow-up completed)	43 (95.5)
Non-response, *n* = (% of follow-up completed)	2 (4.5)
Lost to follow-up, *n* =	1 (excluded from the study)
Persistence of HCV infection: *n* = (%)	6 (10)
HIV coinfection: *n* = (%)	51 (85)
Patients on ART: *n* = (%)	38 (74.5)
CD4 count, cells/µL: mean (range)	561 (207–1561)
HIV patients with <50 copies/mL: *n* = (% of patients on ART)	32 (62.7)

Abbreviations: ALT: alanine aminotransferase; AST: aspartate aminotransferase; γGT: γ-glutamyl-transferase; INR: international normalized ratio; IL: interleukin; HCV: hepatitis C virus; HIV: human immunodeficiency virus; ART: antiretroviral therapy. * The time point of acute hepatitis C infection was determined according to the medical history or, if not applicable, the time point of infection was estimated by calculating the mean date between the last time point with normal aminotransferases and the first time point with a positive HCV RNA. Missing data: ALT levels were missing in 2 patients; AST levels were missing in 2 patients; γGT levels were missing in 2 patients; bilirubin levels were missing in 3 patients; thrombocyte levels were missing in 11 patients; INR levels were missing in 21 patients.

**Table 2:** Univariate analysis of baseline parameters associated with spontaneous clearance compared to HCV persistence.

Parameters	Spontaneous Clearance (*n* = 11)	Persistence of HCV (*n* = 6)	*p*-Value
Age, years: mean (range)	42 (22–54)	43.5 (37–56)	0.2
ALT, IU/L: mean (range)	437 (113–2217)	291.5 (43–922)	0.4
AST, IU/L: mean (range)	224 (51–745)	132 (33–402)	0.1
γGT, IU/L: mean (range)	220 (31–310)	205.5 (93–407)	0.7
Bilirubin, mg/dL: mean (range)	1.35 (0.5–2.2)	0.765 (0.5–2.64)	0.4
Thrombocytes, cells/µL: mean (range)	202.5 (145–307)	209 (153–255)	1.0
INR: mean (range)	1.0 (0.9–1.1)	0.9 (0.9–1.2)	0.6
HCV genotype (*n* =, 1/non-1)	6/5	6/0	0.056
HCV viral load, IU/mL: mean (range)	1.4 × 10^6^ (2780–(6.9 × 10^7^))	1.2 × 10^6^ (64,900–(8.6 × 10^7^))	0.8
HIV coinfection: (*n* =, yes/no)	8/3	6/0	0.1
HIV patients with <50 copies/mL: (*n* =, yes/no)	5/3	5/1	0.4
CD4 count, cells/µL: mean (range)	564.7 (312–850)	480 (207–766)	0.5
*IL28B-rs12979860* genotype: (CC/non_CC, *n* = (%))	5/6	1/5	0.2
C24Cer, ng/mL: mean (range)	1710 (626–2270)	1815 (808–2980)	0.4
C24DHC, ng/mL: mean (range)	88.4 (49.8–138)	173.5 (61.5–258)	0.047
C16Cer, ng/mL: mean (range)	417 (218–743)	459 (310–534)	0.8
C16DHC, ng/mL: mean (range)	119 (57.1–239)	171.5 (116–244)	0.07
Sphingosine, ng/mL: mean (range)	13.3 (5.7–37.8)	11.1 (5.5–15.6)	0.6
Sphinganine, ng/mL: mean (range)	2.96 (1.75–6.71)	2.83 (1.74–4.49)	0.6
S1P, ng/mL: mean (range)	424 (359–698)	511 (244–648)	0.8
dhS1P, ng/mL: mean (range)	50.7 (35.3–75.3)	61.6 (15.6–96.6)	0.8

Abbreviations: ALT: alanine aminotransferase; AST: aspartate aminotransferase; γGT: γ-glutamyl-transferase; INR: international normalized ratio; IL: interleukin; HCV: hepatitis C virus; HIV: human immunodeficiency virus; DHC: dihydroceramide; Cer: ceramide; S1P: sphingosine 1-phosphate; dhS1P: dihydrosphingosine 1-phosphate. Missing data: bilirubin levels were missing in 1 patient with spontaneous clearance of HCV; thrombocyte levels were missing in 3 patients with spontaneous clearance of HCV; INR levels were missing in 3 patients with spontaneous clearance and 3 patients with persistence of HCV.

## Supplementary Materials

Supplementary materials can be found at http://www.mdpi.com/1422-0067/17/6/922/s1.

## Abbreviations

HCV	hepatitis C virus
IDU	injecting drug users
MSM	men having sex with men
HIV	human immunodeficiency virus
DAA	direct-acting antiviral
SL	sphingolipid
ANGPTL3	angiopoietin-like 3
Cer	ceramide
DHC	dihydroceramide
ALT	alanine aminotransferase
SVR	sustained viral response
LC-MS/MS	liquid chromatography-tandem mass spectrometry
S1P	sphingosine 1-phosphate
dhS1P	sphinganine 1-phosphate
APCI	atmospheric pressure chemical ionization
ESI	electrospray ionization
MRM	multiple reaction monitoring
CI	confidence interval
PEG-IFN	PEGylated interferon
RBV	ribavirin
INR	international normalized ratio
