# Supplementary material for: Persistence of HCV in Acutely-Infected Patients Depletes C24-Ceramide and Upregulates Sphingosine and Sphinganine Serum Levels
Source: Int J Mol Sci. 2016 Jun 13;17(6):922. doi: 10.3390/ijms17060922 (PMC4926455; doi:10.3390/ijms17060922)
Supplement: Supplementary file 1 [file ijms-17-00922-s001.pdf]

Supplementary Methods: Persistence of HCV in Acutely-Infected Patients Depletes C24-Ceramide and Upregulates Sphingosine and Sphinganine Serum Levels

Georgios Grammatikos, Julia Dietz, Nerea Ferreiros, Alexander Koch, Georg Dultz, Dimitra Bon, Ioannis Karakasiliotis, Thomas Lutz, Gaby Knecht, Peter Gute, Eva Herrmann, Stefan Zeuzem, Penelope Mavromara , Christoph Sarrazin and Josef Pfeilschifter

Determination of Sphingolipid Concentrations by High-Performance Liquid Chromatography Tandem Mass Spectrometry

A Luna C18 column (150 × 2 mm ID, 5 µm particle size, 100 Å pore size; Phenomenex, Aschaffenburg, Germany) was used for chromatographic separation. The HPLC mobile phases consisted of water:formic acid (100:0.1, *v*/*v*) (A) and acetonitrile:tetrahydrofuran:formic acid (50:50:0.1, *v*/*v*/*v*) (B). For separation, a gradient program was used at a flow rate of 0.3 mL/min. The initial buffer composition 60% (A)/40% (B) was hold for 0.6 min and then in 3.9 min linearly changed to 0% (A)/100% (B) and hold for 6.5 min. Subsequently the composition was linearly changed within 0.5 min to 60% (A)/40% (B) and then held for another 4.5 min. The running time for every sample (injection volume: 15 µL for Cer and dihydroceramides (DHCs) determination and 10 µL for the other sphingolipids) was 16 min. MS/MS analyses were performed on a API4000 (triple quadrupole mass spectrometer) equipped with an APCI (Atmospheric Pressure Chemical Ionization) ion source (AB Sciex, Darmstadt, Germany) for Cer and DHC determination, and with an ESI (Electrospray Ionization) ion source for sphingosine, sphinganine and their 1-phosphate derivatives determination. The analysis was done in Multiple Reaction Monitoring (MRM) mode. For every analyte two transitions were recorded: one for quantification and another one for qualification, to exclude false positive results, with a dwell time of 50 ms. For analysis and quantification the Analyst Software 1.5 (AB Sciex, Darmstadt, Germany) was used and the peak area of the analyte was corrected by the peak area of the internal standard. Linearity of the calibration curve was proven for C16:0Cer, C24:0Cer, C16:0DHC, C24:1DHC, C24:0DHC from 0.6 to 1.000 ng/mL, for C18:0Cer from 0.18 to 300 ng/mL, for C20:0Cer, C24:1Cer from 0.24 to 400 ng/mL and for C18:0DHC from 0.3 to 500 ng/mL. For sphingosine, S1P and SA1P the calibration curve ranged from 0.15 to 250 ng/mL. The coefficient of correlation was at least 0.99. Variations in accuracy were less than 15% over the whole range of calibration.

Table S1. Correlation of baseline serum sphingolipids with age, viral load, biochemical markers and angiopoietin-like 3 (ANGPTL3) in patients with acute hepatitis C virus (HCV) infection. * $p < 0.05$, ** $p < 0.01$, *** $p < 0.001$.

SL	Age	ALT	AST	γGT	Bilirubin	Viral Load	ANGPTL3
C16DHC	0.150	0.103	0.119	0.084	0.230	0.025	0.047
C18DHC	0.132	0.152	0.154	0.204	0.226	−0.036	0.111
C24DHC	0.469 ***	−0.265 *	−0.265 *	−0.180	−0.198	−0.091	−0.249
C24:1DHC	0.200	0.153	0.174	0.146	0.08	0.004	−0.018
C16Cer	−0.018	0.274 *	0.236	0.373 **	0.240	−0.081	0.233
C18Cer	−0.059	0.294 *	0.268 *	0.384 **	0.302 *	0.045	0.265 *
C20Cer	0.125	0.260 *	0.255	0.419 **	0.226	0.040	0.228
C24Cer	0.386 **	−0.257	−0.315 *	−0.084	−0.328 *	−0.129	−0.198
C24:1Cer	0.112	0.274 *	0.265 *	0.323 *	0.253	0.041	0.161
Sphingosine	0.145	0.294 *	0.248	0.356 **	0.171	−0.131	0.201
Sphinganine	0.024	0.197	0.180	0.183	0.161	−0.092	0.204
S1P	0.360 **	0.100	0.069	0.168	0.121	−0.286 *	0.034
dhS1P	0.198	0.161	0.158	0.124	0.010	−0.154	0.007

Correlation Is Evaluated by Spearman's Correlation Coefficient Rho (r)

Abbreviations: ALT: alanine aminotransferase, AST: aspartate aminotransferase, γGT: γ-glutamyl-transferasε, DHC: dihydroceramide, Cer: ceramide, S1P: sphingosine 1-phosphate, dhS1P: dihydrosphingosine 1-phosphate. Significant correlations are shown in bold and are indicated in the corresponding Figures: * $p < 0.05$, ** $p < 0.01$, *** $p < 0.001$.

Missing data: ALT levels were missing in 2 patients, AST levels were missing in 2 patients, γGT levels were missing in 2 patients, Bilirubin levels were missing in 3 patients.

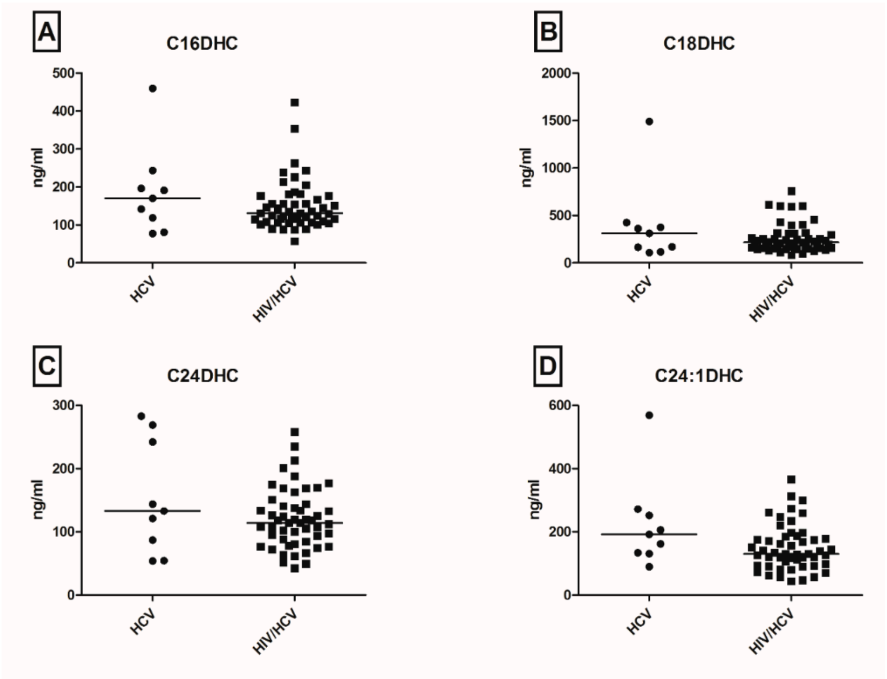

Figure S1. Serum dihydroceramide levels in hepatitis C virus (HCV)-monoinfection and in HCV/human immunodeficiency virus (HIV)-coinfection. No significant differences in the levels of distinct DHC's were observed between mono- and coinfected patients. DHC: dihydroceramide.

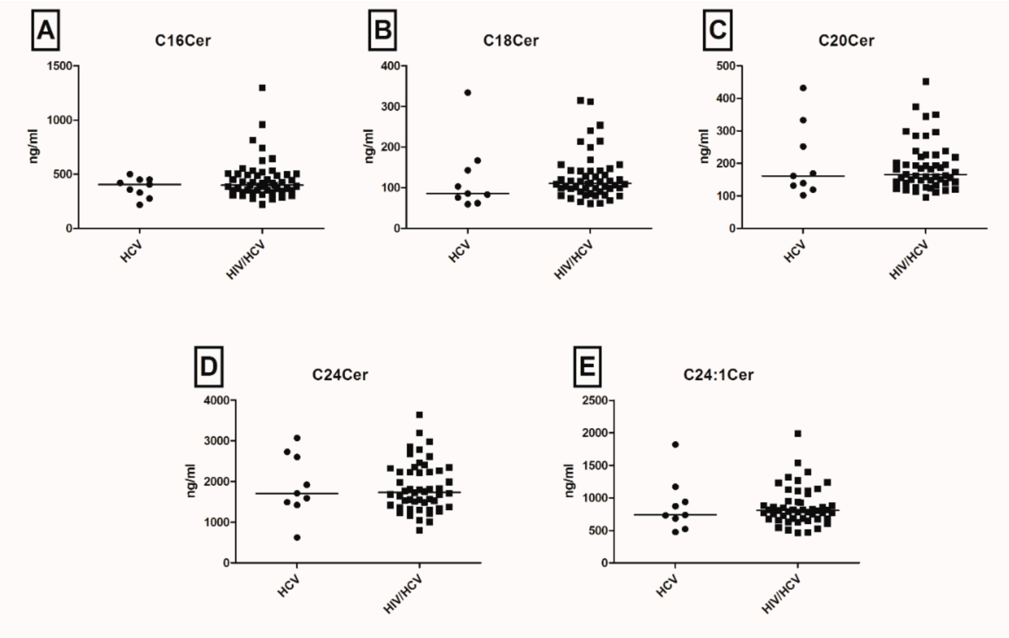

Figure S2. Serum ceramide levels in HCV-monoinfection and in HCV/HIV-Coinfection. No significant differences in the levels of distinct Cer's were observed between mono- and coinfected patients. Cer: ceramide.

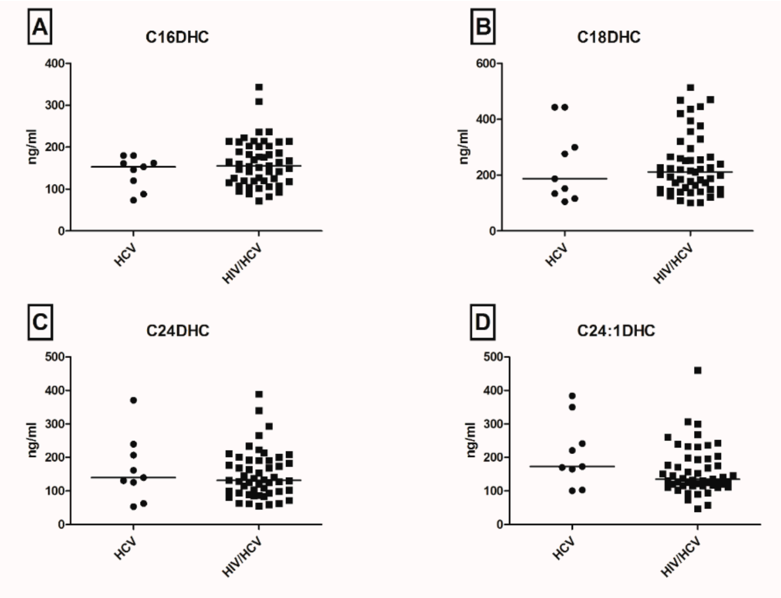

Figure S3. Serum dihydroceramide levels in HCV-mono-infection and in HCV/HIV-Coinfection at follow up. No significant differences in the levels of distinct DHC's were observed between mono- and co-infected patients. DHC: dihydroceramide.

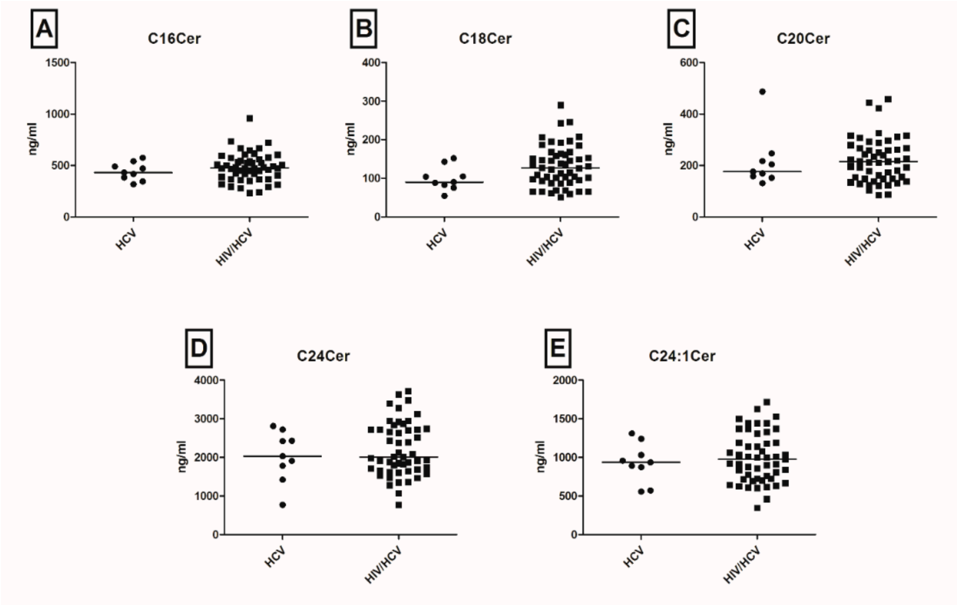

Figure S4. Serum ceramide levels in HCV-mono-infection and in HCV/HIV-Coinfection at follow up. No significant differences in the levels of distinct Cer's were observed between mono- and co-infected patients on follow up. Cer: ceramide.

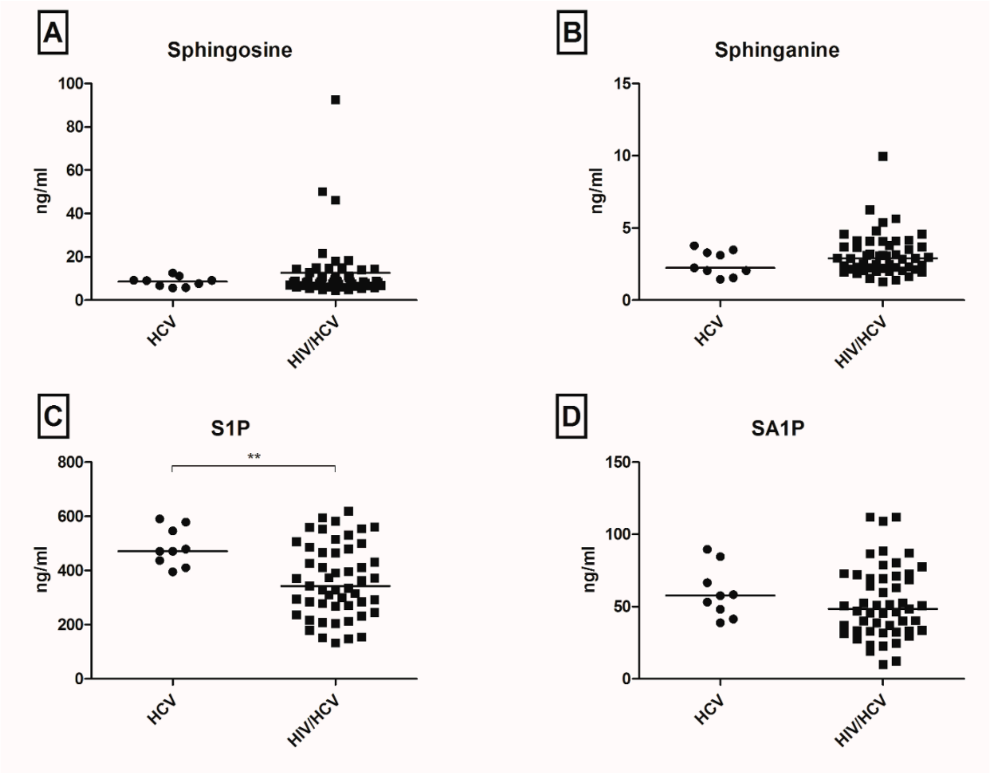

Figure S5. Serum SL's and ANGPTL3 in HCV-mono-infection and in HCV/HIV.

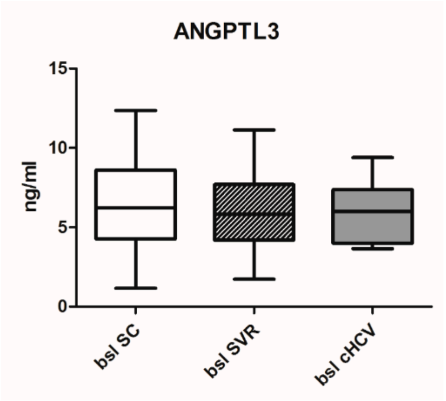

Figure S6. Serum ANGPTL3 levels among patient groups. No significant differences in the baseline levels of ANGPTL3 were observed between patients with spontaneous clearance, HCV persistence and patients with SVR after subsequent antiviral therapy. ANGPTL3: angiopoietin-like 3 protein, bsl: baseline, SC: spontaneous clearance, SVR: sustained viral response, cHCV: chronic HCV.

Co-infection at follow up. S1P levels in HCV/HIV-co-infected patients were lower than in HCV-monoinfected patients (6C, ** $p < 0.01$) at baseline. No significant differences in the levels of sphingosine and sphinganine were observed among mono- and co-infected patients. S1P: sphingosine 1-phosphate, SA1P: sphinganine 1-phosphate.
